# Folate Receptor Alpha Autoantibodies in Early Pregnancy: First-Trimester Reference Intervals and Proposed Clinical Thresholds

**DOI:** 10.3390/mps9030079

**Published:** 2026-05-25

**Authors:** Claudio Giorlandino, Marina Cupellaro, Katia Margiotti, Francesca Giorlandino, Francesco Pignataro, Maria Luisa Mastrandrea, Raffaella Raffio, Laura D’Emidio, Alvaro Mesoraca, Vincenzo Milite

**Affiliations:** 1 Department of Prenatal Diagnosis, Fetal-Maternal Medical Centre, Altamedica, Viale Liegi 45, 00198 Rome, Italy; claudio.giorlandino@artemisia.it (C.G.); francesco.pignataro@artemisia.it (F.P.); marialuisamastrandrea@yahoo.it (M.L.M.); raffaella.raffio@artemisia.it (R.R.); laura.demidio@artemisia.it (L.D.); vinmilite@hotmail.com (V.M.); 2 Department of Clinical Biochemistry and Laboratory Medicine, Altamedica, Viale Liegi 45, 00198 Rome, Italy; marina.cupellaro@artemisia.it (M.C.); francesca.giorlandino@artemisia.it (F.G.); 3 Human Genetics Laboratory, Altamedica, Viale Liegi 45, 00198 Rome, Italy; alvaro.mesoraca@artemisia.it

**Keywords:** folate receptor alpha autoantibodies, first-trimester pregnancy, placental folate transport

## Abstract

Maternal folate receptor alpha autoantibodies (FRAA) have been associated with impaired placental folate transport, fetal cerebral folate deficiency (CFD) and neurodevelopmental risks including autism spectrum disorder (ASD); however, first-trimester-specific reference intervals remain undefined. This prospective single-center study of 534 healthy pregnant women at 10 + 0 to 15 + 6 weeks’ gestation (week 10 n = 26; week 11 n = 155; week 12 n = 203; week 13 n = 105; week 14 n = 39; week 15 n = 6) used a CE-IVDR FRAA ELISA, following CLSI EP28-A3c and IFCC C-RIDL protocols, to establish week-specific percentiles (P5, P50, P95, P99) via non-parametric estimation and log-smoothed regression with a 1-week rolling window. An internal-consistency ROC analysis was performed against the within-dataset ≥P99 designation and is therefore not interpretable as discrimination against an independent clinical outcome. Median FRAA declined from 29 ng/mL (week 10) to 25 ng/mL (week 14), with provisional clinically actionable thresholds of P95 ≈ 120 ng/mL and P99 ≈ 150 ng/mL. These data provide the first first-trimester normative percentile curves for maternal FRAA and may support prioritizing FRAA assessment before 15 weeks (onset of accelerated transplacental IgG transfer). Given the cross-sectional, single-center design, the small samples at weeks 14–15, and the absence of long-term neurodevelopmental outcome data, the proposed thresholds and any downstream clinical implications, including folinic acid intervention and ASD risk mitigation, should be considered hypothesis-generating and require external and longitudinal validation.

## 1. Introduction

Folate metabolism plays a critical role in fetal brain development, neurogenesis, synaptic maturation, and epigenetic programming [1,2,3,4]. Disrupted folate transport across the placenta, particularly due to maternal FRAA, can impair neuronal folate availability, contributing to CFD and increased risk of ASD [5].

While FRAA prevalence is well-documented in children with CFD and ASD, first-trimester maternal reference ranges remain undefined. This gap is critical, as transplacental IgG transfer accelerates from 13 to 15 weeks gestation, enabling pathogenic FRAA to reach the fetus during peak neurodevelopment [6,7,8]. Standard prenatal folic acid reduces neural tube defects but, on the basis of preclinical and clinical reports, may be insufficient in FRAA-positive pregnancies because of receptor blockade. Folinic acid may bypass this block via alternative transporters such as the reduced folate carrier (RFC). Preliminary pilot data suggest that folinic acid supplementation might be associated with a lower ASD incidence (10% vs. 62.5%) and with improved cognitive outcomes in FRAA-positive pregnancies, but these findings derive from small, non-confirmatory studies and should not be regarded as proof of efficacy [9]. The objective of the present study is therefore to provide the first structured reference percentile curves for maternal FRAA in the first trimester and to propose provisional clinical thresholds for immune-mediated folate deficiency risk stratification, to be validated in future prospective outcome-based studies.

## 2. Methods

### 2.1. Study Design and Population

This prospective reference-interval study enrolled 534 healthy pregnant women at 10 + 0–15 + 6 weeks’ gestation, distributed across gestational weeks according to routine clinical availability as follows: week 10 (n = 26), week 11 (n = 155), week 12 (n = 203), week 13 (n = 105), week 14 (n = 39) and week 15 (n = 6); aggregated, the 11–13 week stratum comprised 463 women. We note that the sample sizes available at weeks 14 and 15 are below the CLSI EP28-A3c minimum of 120 observations recommended for stable non-parametric estimation of extreme upper percentiles; consequently, percentile estimates at these gestational weeks should be regarded as exploratory and were retained for descriptive completeness only. The study was approved by the Artemisia spa ethical committee and conducted per the 1964 Declaration of Helsinki. Inclusion criteria: viable singleton pregnancy confirmed by ultrasound, gestational age 10 + 0–15 + 6 weeks via crown–rump length, maternal age 18–42 years, ability to provide informed consent, no known obstetric complications. Exclusion criteria: pre-existing autoimmune diseases (systemic lupus erythematosus, rheumatoid arthritis, antiphospholipid syndrome, inflammatory bowel disease, type 1 diabetes, thyroid autoimmunity); systemic immunosuppressants/immunomodulators (>10 mg/day prednisone equivalent, methotrexate, biologics); folic acid > 1 mg/day or high-dose folinic acid; multiple gestation; major fetal anomalies/aneuploidy; CKD stage ≥ 3, active malignancy, organ transplant, chronic viral infections (HIV, HBV, HCV); tobacco (>5 cigarettes/day) or substance abuse. Although clinically silent inflammatory or autoimmune conditions could theoretically still influence circulating FRAA levels, all participants were screened by standardized obstetric history and routine first-trimester laboratory work-up at enrolment, and none exhibited clinical or biochemical signs of acute or chronic inflammation. Maternal serum folate, vitamin B12, homocysteine, MTHFR genotype, body mass index, periconceptional supplementation compliance, dietary habits and socioeconomic indicators were not systematically captured in this dataset; these potential confounders are acknowledged among the study limitations and should be measured in future validation cohorts. No major fetal anomalies were detected at the time of enrolment, but the cross-sectional design did not include follow-up of offspring for neurodevelopmental outcomes (including ASD), which is acknowledged as an important limitation.

### 2.2. Sample Collection and Processing

Peripheral blood (5 mL K3-EDTA) was collected after overnight fasting and centrifuged (2000× *g*, 10 min, 4 °C) within 2 h, and separated plasma was stored at −80 °C until analysis (stability verified 6 months, 5 freeze–thaw cycles, recovery >95%). Folate receptor alpha autoantibody (FRAA) concentrations were quantified using the commercial CE-IVDR sandwich ELISA kit (Altamedica Diagnostics, Rome, Italy; www.altamedica.it; accessed on 20 May 2025); sensitivity LoD 3.2 ng/mL on 120 blanks; reportable range 7.8–500 ng/mL, 1:5 plasma dilution in PBS 0.05% Tween20). Analytical validation (n = 534 samples + QC): Intra-assay CV 3.8% (low QC 15 ng/mL, 20 replicates), inter-assay CV 6.2% (5 days), linearity r^2^ = 0.998 (serial dilutions 10–400 ng/mL), recovery 98–102%, no significant interference (hemolysis/lipemia/icterus index <5%). Calibration via 4-parameter logistic (4PL) standard curve, R^2^ > 0.99 per plate. Positive/negative agreement was 98% vs. Western blot (n = 50 controls).

### 2.3. Reference Interval Construction

Reference values were determined according to CLSI EP28-A3c and IFCC C-RIDL international harmonization protocols [10,11]. Percentiles P5, P50, P95, and P99 were calculated per gestational week using non-parametric methods; log transformation addressed skewness; and smoothed curves generated via rolling-window regression (1-week window) per IFCC recommendations for extreme percentile stabilization.

### 2.4. Statistical Analysis

The distribution of the variables was assessed for normality using the Shapiro–Wilk test; given the right-skewness typical of immunoassay data, log transformation [ln(FRAA + 1)] was applied for smoothing as detailed in Section 2.3. Sample-size adequacy for non-parametric percentile estimation was evaluated according to the CLSI EP28-A3c framework. With a total of n = 534 and an aggregated 10–13 week stratum of n = 489, the dataset exceeds the CLSI minimum of 120 observations for non-parametric estimation of P95 and P99 in the well-populated gestational windows, whereas the partitioned strata at weeks 14 (n = 39) and 15 (n = 6) fall below this threshold and are presented for descriptive purposes only. As an internal-consistency check, a ROC curve was also constructed using the within-dataset ≥P99 designation as the positive label. Because the positive label is derived from the same distribution that defines the percentile cut-off, the resulting area under the curve (AUC) reflects the internal mathematical separability of the data around the cut-off and not discrimination against an independent clinical, histopathological or neurodevelopmental endpoint. The AUC value should therefore be interpreted as a quality-control verification that escalating FRAA concentrations correctly rank against the percentile-derived threshold, and not as evidence of diagnostic performance against a true clinical outcome. A formal outcome-based ROC analysis will require an external validation cohort with predefined clinical endpoints.

## 3. Results

**Figure 1:**
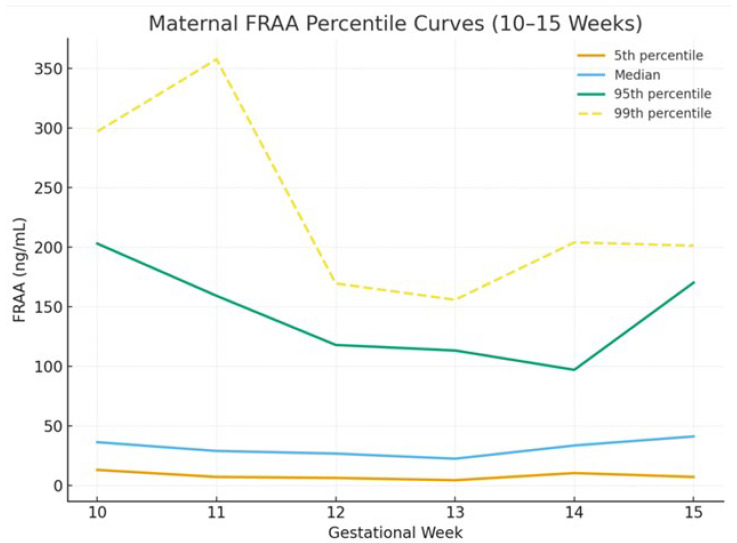
Raw Percentile Curves (P5, P50, P95, P99). This figure depicts gestational-age-specific percentile curves for maternal folate receptor alpha autoantibodies (FRAA) from 10 to 15 weeks’ gestation. Percentiles were calculated using a non-parametric method following CLSI EP28-A3c and IFCC reference interval guidance. The 5th percentile represents the lower boundary of expected physiologic variation, the median (50th percentile) indicates the central tendency, and the 95th and 99th percentiles define the upper limits of the reference interval and the proposed pathological decision threshold, respectively. A progressive physiological decline in median FRAA values is observed across gestation, consistent with evolving maternal–fetal immune modulation and folate transport equilibrium. Extreme upper-tail values above the 99th percentile are interpreted, on the basis of the published literature, as potentially impairing placental folate transport. Note: percentile estimates at week 14 (n = 39) and week 15 (n = 6) are below the CLSI EP28-A3c recommended minimum sample size of 120 per stratum for stable upper-percentile estimation and are presented for descriptive purposes only.

Figure 1 illustrates the gestational age-specific percentile distribution of maternal FRAA concentrations (10 + 0–15 + 6 weeks), showing raw non-parametric curves (P5, P50, P95, P99) and the physiological median decline from ~29 ng/mL (week 10) to ~25 ng/mL (week 14). Table 1 reports week-specific percentiles (P5, P50, P95, P99) with two-decimal precision, derived via non-parametric methods per CLSI EP28-A3c and IFCC C-RIDL guidelines [9,10,11,12], partitioned by gestational week as physiological covariate. These constitute the first proposed trimester-specific FRAA reference percentiles: P95 (≈120 ng/mL) and P99 (≈150 ng/mL) are proposed as provisional borderline and pathological cut-offs, respectively. Owing to the imbalanced distribution across gestational weeks, percentile estimates for weeks 14 and 15 (n = 39 and n = 6) should be interpreted with caution and are presented for descriptive purposes only; the stable estimates for clinical use derive from the well-populated 10–13-week window (n = 489).

**Figure 2:**
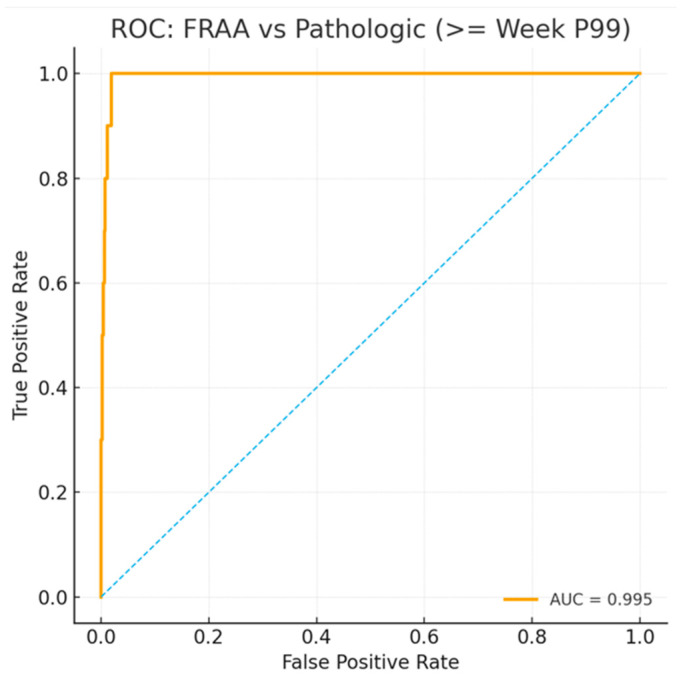
ROC Curve (Pathological ≥ Week-Specific P99) The ROC curve was constructed as an internal consistency check using the within-dataset ≥P99 designation as the positive label, defined according to CLSI EP28-A3c decision-limit methodology. The area under the curve (AUC = 0.995) reflects the internal mathematical separability of FRAA concentrations around the percentile-derived cut-off rather than diagnostic discrimination against an independent clinical, histopathological or neurodevelopmental endpoint. This analysis should therefore be interpreted as quality-control verification of monotone ranking against the percentile threshold; formal validation of FRAA as a diagnostic biomarker of disrupted folate transport or fetal cerebral folate deficiency requires independent outcome-based cohorts.

As an internal consistency check, the relationship between FRAA concentrations and the within-dataset ≥P99 designation is depicted in Figure 2 (ROC analysis). The resulting AUC of 0.995 confirms internal mathematical separability around the percentile-derived cut-off, but should not be interpreted as discriminatory performance against an independent clinical or neurodevelopmental endpoint (see Methods, Section 2.4).

**Figure 3:**
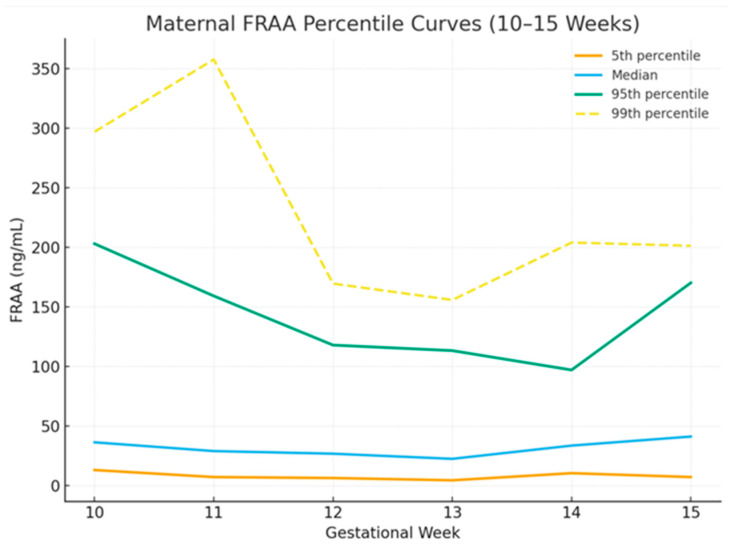
Smoothed Log-Transformed Percentile Curves. Log transformation [ln (FRAA + 1)] was applied to mitigate right-skewness and stabilize variance characteristics of immunoassay-derived biomarkers. Smoothed percentile curves were generated using a gestational week rolling window (±1 week) consistent with IFCC recommendations for non-parametric centile smoothing. The 5th, 50th, and 95th percentiles demonstrate a graded decline across gestation, indicating progressive downregulation of maternal autoantibody expression during early placental development. Log-scale modelling improved curve smoothness, precision, and biological plausibility, particularly at upper percentiles where extreme values cluster.

**Figure 4:**
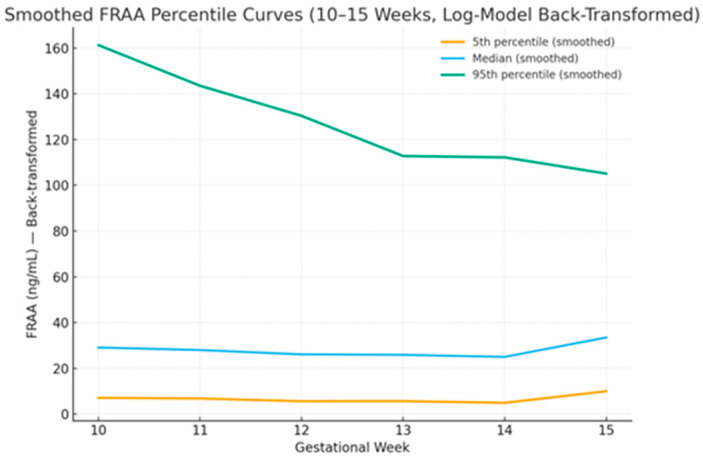
Back-Transformed Smoothed Percentile Curves. Smoothed log-transformed percentiles were exponentiated to return values to the clinical ng/mL scale. The resulting smoothed curves preserve statistical advantages of log transformation while providing clinically interpretable values. Median FRAA values decline from approximately 29 ng/mL at 10 weeks to ~25 ng/mL at 14 weeks, whereas upper-tail values (>P95) remain markedly elevated in early gestation before tapering, consistent with dynamic maternal immune modulation. These back-transformed reference values represent the first pregnancy-specific normative ranges for FRAA in the literature.

According to published mechanistic and clinical literature (the present study did not perform original functional or mechanistic experiments), folate receptor-alpha (FRα), the primary high-affinity transporter for 5-methyltetrahydrofolate across the syncytiotrophoblast, functions during a critical developmental window of maximal DNA synthesis, one-carbon metabolism, phospholipid production, and epigenetic programming demands [13]. Elevated FRAA levels have been described as impairing folate transport via three convergent mechanisms reported in prior studies: competitive binding to FRα causing steric hindrance and reduced folate translocation into trophoblasts, antibody-induced receptor internalization with lysosomal degradation that lowers surface FRα despite adequate extracellular folate, and complement-activating FRAA subclasses triggering placental microinjury and oxidative stress. In the published literature, these mechanisms have been associated with downstream effects including impaired methylation capacity, heightened oxidative stress, disrupted neuronal migration, and hypomyelination; the present reference-interval study did not generate original data on any of these downstream endpoints. From a clinical timing perspective, accelerated maternal IgG transfer via FcRn initiates at 13–15 weeks [6,7], providing a rationale for considering FRAA assessment before transplacental antibody passage. Our smoothed percentile curves (Figure 3 and Figure 4) document the gestational distribution of FRAA in healthy first-trimester pregnancies and, when interpreted in the context of the published literature on FcRn-mediated transfer, define an early window in which elevated maternal autoantibodies might exert their effect prior to substantial fetal exposure; demonstrating that such elevations causally impair folate transport in this cohort was not within the scope of the present study.

Important caveat: the present study did not perform functional, biochemical or histological analyses on placental or fetal tissue, nor did it follow up offspring for neurodevelopmental outcomes. The biochemical and neurodevelopmental considerations summarized below are therefore drawn entirely from the prior experimental and clinical literature and are presented as a literature-based mechanistic framework, not as original findings of this work. In previous studies, FRAA-mediated disruption of placental folate transport has been associated with reduced methyl donor availability and DNA hypomethylation during key epigenetic reprogramming phases, and with elevated oxidative stress markers and mitochondrial dysfunction in fetal/neural models, reflecting cellular energy deficits from folate deficiency.

Neurodevelopmentally, previous studies have reported that these biochemical alterations may manifest as disrupted axonal guidance, neural tube patterning defects, inefficient synaptogenesis, disorganized neuronal migration, and delayed white-matter maturation indicative of early hypomyelination. Taken together with such published mechanistic and clinical evidence, the temporal placement of FRAA assessment before substantial IgG transfer suggests a biologically plausible window for screening and potential intervention [1,2,3,4,5,6,7,8,9]; however, the present study, as a cross-sectional reference-interval study, does not itself demonstrate this causal pathway in our cohort.

## 4. Discussion

The present study addresses an unmet need in perinatal laboratory medicine by proposing, for the first time, trimester-specific reference percentiles for maternal folate receptor alpha autoantibodies (FRAAs) in early pregnancy. In the published literature, maternal FRAAs have been implicated in cerebral folate deficiency (CFD) and in an increased risk of neurodevelopmental disorders, including autism spectrum disorder (ASD), primarily through disruption of folate transport to the developing brain [1,2,3,4,5,6,7,8,9]. However, previous work has largely focused on affected children or on retrospective maternal samples, without offering a structured, prospective framework to interpret FRAA concentrations during the first trimester. We wish to make clear from the outset that the present work is a reference-interval study, designed and reported in accordance with the CLSI EP28-A3c and IFCC C-RIDL recommendations [10,11]; it does not directly address clinical, mechanistic or neurodevelopmental outcomes, all of which are extrapolated from prior literature and must therefore be interpreted as hypothesis-generating rather than as findings of this study. With this important caveat, our gestational week-specific percentiles document a progressive physiological decline in median FRAA values from approximately 29 ng/mL at 10 weeks to approximately 25 ng/mL at 14 weeks, while upper percentiles (P95, P99) remain elevated in a subset of women. Read together with the published mechanistic literature, this pattern is consistent with the hypothesis of an “immune vulnerability window” during which heightened autoantibody activity could, in principle, exert effects on placental folate transport; demonstration of such an effect in our cohort is beyond the design of this study. The provisional stratification model translates these reference data into a pragmatic interpretative framework: FRAA levels below 120 ng/mL (P95) appear compatible with a physiological immune profile and standard prenatal follow-up and concentrations between 120 and 150 ng/mL may indicate a borderline state of immune activation that could warrant repeat testing and optimization of maternal nutritional and metabolic support, whereas values exceeding 150 ng/mL (P99) identify a clearly outlying autoantibody profile. These cut-offs are broadly consistent with immunopathogenic ranges previously reported in CFD and ASD pediatric and maternal cohorts, supporting their biological plausibility [14]. The mechanistic considerations discussed below are summarized from prior experimental and clinical literature and were not investigated in the present cohort. Previous studies have reported that FRAA can impair folate delivery to the fetal compartment through multiple convergent mechanisms; autoantibodies directed against FRα, the primary high-affinity transporter for 5-methyltetrahydrofolate across the syncytiotrophoblast, may competitively block ligand binding, promote receptor internalization and degradation, and, in specific subclasses, activate complement pathways leading to placental microinjury and oxidative stress. In animal and pediatric models of CFD and in children with FRAA-associated ASD, these mechanisms have been associated with reduced methylation capacity, increased oxidative stress and signatures of impaired neuronal migration and myelination. Our smoothed percentile curves, showing elevated upper-tail FRAA concentrations early in gestation with a subsequent decline, are compatible with this published mechanistic framework but do not, by themselves, demonstrate fetal or placental effects in our cohort. The timing of testing is a relevant clinical dimension. Active transport of maternal IgG via the neonatal Fc receptor (FcRn) accelerates around 13–15 weeks of gestation; on this basis, FRAA assessment performed at or before 15 weeks would, in principle, maximize the opportunity to identify women with outlying autoantibody profiles before substantial transplacental antibody transfer occurs. This pre-transfer window coincides with peak demands for folate-dependent processes such as DNA synthesis, one-carbon metabolism, phospholipid biosynthesis and epigenetic programming in the fetal nervous system. Within this temporal context, our reference framework may offer a prospective tool to identify potentially at-risk pregnancies earlier than previously feasible. Any subsequent decision regarding targeted intervention, such as folate-pathway optimization, folinic acid supplementation in FRAA-positive mothers or closer neurodevelopmental surveillance of the offspring, should however be considered exploratory pending outcome-based confirmation. From a clinical perspective, the proposed thresholds may support a tiered observational approach to care, in the context of clinical studies and not as routine practice: women with FRAA concentrations in the physiological range can continue routine prenatal management, while those in the borderline interval might be considered for repeat testing to assess trajectory and for careful evaluation of dietary folate intake, vitamin B12 status and other one-carbon metabolism co-factors. For FRAA levels above 150 ng/mL (P99), early initiation of targeted folate-pathway support and multidisciplinary follow-up could be discussed within a research setting, given the concordance with immunopathogenic thresholds reported in CFD and ASD cohorts; however, in the absence of prospective outcome data linking these specific thresholds to clinical endpoints, we explicitly recommend against using them as decision rules in routine clinical practice at this time. Several aspects of our work strengthen its potential impact. First, the study adheres to international standards for reference interval definition (CLSI EP28-A3c, IFCC C-RIDL), including rigorous pre-analytical control, exclusion of conditions associated with immune activation, and a sufficient aggregated sample size across the 10–13-week window to support stable non-parametric percentile estimation. Second, the FRAA assay used is CE-IVDR-validated, with documented analytical performance characteristics (sensitivity, reportable range, imprecision, linearity and lack of major interferences), meeting contemporary expectations for clinical implementation of novel biomarkers. Third, the modelling approach, combining log transformation, non-parametric percentile estimation and smoothed curves across gestational weeks, balances statistical rigor with clinical interpretability, providing both tabulated cut-offs and visual tools for risk stratification. Several important limitations must, however, be acknowledged. First, the distribution of participants across gestational weeks is markedly imbalanced, with only 39 women at week 14 and 6 women at week 15—sample sizes well below the CLSI EP28-A3c minimum of 120 observations recommended for stable non-parametric estimation of extreme upper percentiles. P95 and especially P99 estimates at these weeks are therefore inherently unstable and may be driven by individual observations; for any clinical interpretation, we recommend using the aggregated 10–13-week stratum (n = 489), which exceeds CLSI minimum requirements, and we retained week-14 and week-15 percentiles for descriptive completeness only. Second, the ROC analysis presented in Figure 2 was performed using a within-dataset ≥P99 designation as the positive label and therefore reflects internal mathematical separability around the percentile-derived threshold rather than discrimination against an independent clinical, histopathological or neurodevelopmental endpoint; the high AUC value (0.995) should not be interpreted as evidence of diagnostic accuracy, and a formal outcome-based ROC analysis remains to be performed in independent cohorts. Third, this is a single-center study and the population is predominantly Italian and ethnically homogeneous; periconceptional supplementation habits, dietary patterns and broader socioeconomic indicators in this population may not be representative of other geographical or ethnic groups, limiting external generalizability. Fourth, although our exclusion criteria were extensive, clinically silent inflammatory or autoimmune conditions may not have been fully captured by routine first-trimester screening and could in principle influence circulating FRAA. Fifth, important biological confounders—maternal serum folate, vitamin B12, homocysteine, MTHFR genotype, body mass index, supplementation compliance, dietary patterns and socioeconomic status—were not systematically measured in this dataset and should be incorporated as covariates in future validation studies. Sixth, the cross-sectional design does not capture intra-individual FRAA trajectories during pregnancy; longitudinal sampling at multiple timepoints will be needed to establish whether dynamic changes in FRAA titer carry additional prognostic information. Seventh, the proposed thresholds (120 ng/mL and 150 ng/mL) are derived from this cohort and require prospective external validation in independent multicenter cohorts before they can be considered actionable in clinical practice. Eighth, the present study did not follow up offspring for neurodevelopmental outcomes, including ASD, and therefore cannot itself confirm any clinical implications of the proposed cut-offs; longitudinal pregnancy and offspring follow-up are essential. Finally, the recommendation for routine first-trimester FRAA screening and for early folinic acid intervention should be regarded as hypothesis-generating only; randomized controlled trials with neurodevelopmental endpoints, together with formal cost-effectiveness analyses, are required before such a strategy could be considered for clinical adoption. Future multicenter, longitudinal studies with standardized analytical platforms will be essential to harmonize reference intervals internationally, to validate decision thresholds against hard clinical endpoints, and to clarify whether dynamic changes in FRAA titers carry additional prognostic information. Further research should also systematically evaluate intervention strategies in FRAA-positive pregnancies. Randomized controlled trials comparing folinic acid versus standard folic acid supplementation, different dosing regimens and combined metabolic–immunological approaches (e.g., antioxidant support, modulation of inflammatory pathways) will be needed to define optimal therapeutic algorithms for mothers exceeding the proposed pathological threshold. The integration of FRAA measurement with other biomarkers of folate metabolism, placental function and early neural development may refine risk prediction models and help individualize preventive strategies. Finally, formal cost-effectiveness analyses are warranted to assess whether routine first-trimester FRAA screening, alone or in combination with other markers, provides sufficient clinical and societal benefit to justify its implementation at scale. In conclusion, our data provide the first proposed normative percentile curves for maternal FRAA in early pregnancy and identify provisional clinical thresholds of 120 ng/mL (P95) and 150 ng/mL (P99). These reference data offer a structured framework for the future evaluation of immune-mediated folate transport disruption in the first trimester. However, given the cross-sectional, single-center design, the small samples at weeks 14–15, the internal nature of the ROC analysis, and the absence of long-term neurodevelopmental outcome data, the proposed thresholds and any downstream clinical implications—including folinic acid intervention and potential ASD risk mitigation—must be regarded as hypothesis-generating. External validation in independent multicenter cohorts and longitudinal outcome-based studies are required before FRAA testing can be considered for routine integration into prenatal frameworks. Pending such validation, our results provide a methodological and statistical foundation for the prospective investigation of FRAA as a candidate first-trimester biomarker of immune-folate health in pregnancy.

## Figures and Tables

**Table 1:** Clinically actionable reference thresholds for prenatal FRAA interpretation.

Week	N	P5	P50	P95	P99
10	26	13	36.25	202.95	296.9
11	155	7.1	28.9	159.13	357.64
12	203	6.3	26.7	117.87	169.49
13	105	4.38	22.4	113.22	155.77
14	39	10.35	33.5	96.98	203.95
15	6	7.11	41.1	170.15	201.23

## Data Availability

The original contributions presented in this study are included in the article. Further inquiries can be directed to the corresponding author.
